# 85 Systemic lupus erythematosus in Tunisian children: a case series

**DOI:** 10.1093/rheumatology/keac496.081

**Published:** 2022-10-07

**Authors:** H Rouag, S Zayani, A Akid, T Ghedira, E Sfar, R Hadj Salem, C Chouchane, F Thabet, S Chouchane

**Affiliations:** Department of Pediatrics, Fattouma Bourguiba Hospital, Monastir, Tunisia

## Abstract

**Background:**

Childhood–onset systemic lupus erythematosus (c-SLE) is the prototype of a multisystem, inflammatory, heterogeneous autoimmune condition, characterized by simultaneous or sequential organ involvement. Compared with the adult-onset form, c-SLE have a worse prognosis.

**Objectives:**

To review the epidemiological, and bio-clinical, characteristics of a c-SLE case series.

**Methods:**

The files of patients diagnosed as c-SLE in the pediatrics department of Monastir, Tunisia from January 2004 to March 2022 were reviewed. Mean and standard-deviation were used to express normally-distributed variables, as verified by the Kolmogorov-Smirnov statistical test.

**Results:**

Fourteen patients were collected. Female to male ratio was 6:1. Mean ages at lupus onset and diagnosis were 9.9 ± 1.4 years, [5–13.8 years] and 10.75 ± 2.3 years [6–14 years], respectively. Only two children had a family history of autoimmune disease.

The initial admission was motivated primarily by skin and musculoskeletal manifestations, in 64.3% and 51.7% of cases, respectively. General signs (fever, asthenia) were observed in 35.7% of cases, hematological and gastrointestinal manifestations in 28.6% of cases each. In 3 cases, upper gastric endoscopy was performed prior to admission, in view of abdominal pain and vomiting.

The physical examination noted various abnormalities. Malar rash (50%) and discoid lupus (28.6%) were the most frequent cutaneous manifestations, while skin biopsy was performed in three cases, all in keeping with lupus. The musculoskeletal manifestations were arthralgia (71.4%), arthritis and myositis (14.3%). Hematological manifestations included thrombocytopenia and leukopenia in 4 cases, as well as 3 cases of auto-immune hemolytic anaemia and splenomegaly. Renal manifestations were proteinuria in 7, haematuria in 6, and hypertension in 2 (with renal failure in one of the patients). The renal biopsy that was performed in one subject showed a class 2 lupus nephritis. Pleural effusion was observed in 3, pneumonia in 3, pericarditis in 2, myo-pericarditis in 1 and central nervous system (CNS) lupus in 1.

Relevant results of the laboratory workup are illustrated in the following table:

The formal diagnosis of SLE was established according to the ACR-1997 criteria in 7 cases (50%), the SLICC-2012 in 4 cases (28.6%) and EULAR/ACR-2019 in 3 cases (21.4%). The c-SLE diagnosis was associated with coeliac disease and Hashimoto thyroiditis in two of the subjects respectively.

The therapeutic management was based on corticosteroids in 11 cases, hydroxychloroquine in 3, while cyclophosphamides and immunoglobulin were used for two subjects respectively.

The outcomes were heterogeneous. Among 11 patients with sufficient follow-up, 6 cases of remission and 2 cases of relapse were noted. Major adverse events were not infrequent: one case each of cardiac tamponade, macrophage activation syndrome and severe CNS lupus were observed, all fatal.

**Conclusion:**

Childhood–onset systemic lupus is a challenging disease, both to diagnose and to treat. The development of new criteria of higher specificity and sensitivity has greatly helped identify the incomplete types of lupus and allow for early-stage diagnosis, therefore preventing the serious complications of the disease.

**85 Table 1:** 

Laboratory test	Performed (%)	Abnormal results (%)
Erythrocyte sedimentation rate	14 (100%)	13 (92%)
Antinuclear anti-bodies	14 (100%)	13 (92%)
AntiDNA antibodies	7 (50%)	5 (71%)
AntiSm antibodies	8 (57%)	5 (62%)
Antiphospholipid antibodies	8 (57%)	5 (62%)
Complement	11 (78%)	9 (81%)

